# Psychosoziale Notfallversorgung von Kindern nach hoch belastenden Lebenserfahrungen

**DOI:** 10.1007/s00103-022-03586-z

**Published:** 2022-09-15

**Authors:** Simon Finkeldei, Tita Kern, Susanna Rinne-Wolf

**Affiliations:** 1 AETAS Kinderstiftung, München, Deutschland; 2 Fakultät für Sport- und Gesundheitswissenschaften, Uptown München-Campus D, Technische Universität München, Georg-Brauchle-Ring 60/62, 80992 München, Deutschland

**Keywords:** Krisenintervention, Notfall, Trauma, Familie, Psychologische Erste Hilfe, Crisis intervention, Emergency, Trauma, Family, Psychological first aid

## Abstract

Hoch belastende Lebensereignisse wie die Erfahrung einer lebensbedrohlichen Situation oder das Bezeugen von plötzlichem Tod, schwerer Verletzung oder Suizid stellen für die psychische Verarbeitung eine außergewöhnliche Herausforderung dar. Sie stehen in kausalem Zusammenhang mit dem Risiko, an verschiedenen psychischen und psychosomatischen Traumafolgestörungen zu erkranken. Diesem Wissen folgend, sind die Aufgaben der Psychosozialen Notfallversorgung (PSNV): die Prävention psychosozialer Belastungsfolgen, die Früherkennung weiteren Hilfs- oder Versorgungsbedarfs und die Bereitstellung adäquater Hilfe zur Belastungsverarbeitung. Sind Kinder von einem Notfallereignis betroffen, befinden sich diese aufgrund entwicklungspsychologischer Aspekte in einer anderen Position als erwachsene Betroffene.

Der vorliegende Beitrag beschreibt praxisnah die Besonderheiten in der Notfallversorgung der Zielgruppe Kinder. Dabei geht er anhand ausgewählter Forschungsbefunde aus den Feldern Psychotraumatologie und Psychosoziale Notfallversorgung insbesondere der Frage nach, welche Auswirkungen das Bezugspersonenverhalten auf die kindliche Verarbeitung von Notfallereignissen hat. Entsprechende Folgerungen für die Praxis der PSNV werden gezogen. Darüber hinaus werden allgemeine Empfehlungen zur Akutbetreuung von Kindern vorgestellt und Herausforderungen in der Praxis diskutiert.

Die Psychosoziale Notfallversorgung von Kindern erfordert ein im Vergleich zu der Begleitung von erwachsenen Betroffenen angepasstes Vorgehen. Verhaltenskompetenz und Stabilität der Bezugspersonen haben Einfluss auf die kindlichen Verarbeitungsmöglichkeiten. Abhängig vom Zeitpunkt der Intervention bedarf die PSNV unterschiedlicher Kompetenzen. Der niederschwellige Zugang zu Hilfsangeboten stellt in der Versorgungspraxis eine Herausforderung dar.

## Abstract (en)

Highly stressful life events, such as the experience of a life-threatening situation or witnessing sudden death, serious injury, or suicide, pose an extraordinary challenge for psychological processing. They are causally related to the risk of developing various psychological and psychosomatic trauma disorders. Based on this knowledge, the tasks of psychosocial emergency care are the prevention of psychosocial stress consequences, the early identification of further need for help or care, and the provision of adequate help for coping with stress. If children are affected by an emergency, they are in a different position than adults due to developmental psychological aspects.

This article describes the special features of emergency care for the target group of children in a practical way. Based on selected research findings from the fields of psychotraumatology and psychosocial emergency care, it examines in particular the question of the effects of caregiver behaviour on children’s processing of emergency events. Corresponding conclusions for the practice of psychosocial emergency care are drawn. In addition, general recommendations for the acute care of children are presented and challenges in practice are discussed.

The psychosocial emergency care of children requires an adapted approach compared to the care of adult victims. Behavioural competence and stability of the caregivers have an influence on the child’s ability to cope. Depending on the time of the intervention, psychosocial emergency care requires different competences. Low-threshold access to support services is a challenge in care practice.

## Einleitung

Notfälle wie das Erleben eigener Lebensbedrohung oder Zeugenschaft von Verletzung, Gewalt oder Tod betreffen nicht nur Erwachsene. Mehr als die Hälfte aller Kinder und Jugendlichen werden vor dem Erreichen des Erwachsenenalters mit einem oder mehreren potenziell traumatisierenden Lebensereignissen konfrontiert [1]. Schätzungsweise 10–20 % dieser Kinder entwickeln später eine posttraumatische Belastungsstörung (PTBS; [1, 2]) mit dem Risiko weiterer negativer Entwicklungsfolgen [3, 4]. Neben der PTBS stehen weitere Erkrankungen in enger Verbindung mit Traumaerleben [1]. Wie ein hoch belastendes Ereignis verarbeitet wird, ist von verschiedenen Faktoren abhängig.^1^ Dabei spielen Schutz- und Risikofaktoren vor, aber auch Erfahrungen nach der Notfallsituation eine Rolle [5].

Kinder verarbeiten bedrohliche Notfallereignisse anders als Erwachsene. Dies gilt auch für große Schadenslagen wie etwa bei terroristischen Anschlägen oder Naturkatstrophen [6]. Sie sind bei der Verarbeitung von Erfahrungen in vielfältiger Weise auf ihre Bindungspersonen angewiesen [7]. Da erwachsene Bezugspersonen von Notfällen aber oftmals mitbetroffen sind, stehen diese vor der Herausforderung, den Kindern gleichzeitig zur eigenen Verarbeitung einen stabilen Orientierungspunkt zu bieten und sie mit ihren in dieser Situation verfügbaren individuellen Kompetenzen zu unterstützen.

Der vorliegende Beitrag geht anhand ausgewählter Forschungsbefunde aus den Feldern Psychotraumatologie und Psychosozialer Notfallversorgung (PSNV) zunächst der Frage nach, welche Auswirkungen das Bezugspersonenverhalten auf die kindliche Verarbeitung von Notfallereignissen hat und welche Implikationen sich daraus für die PSNV ergeben. Im zweiten Teil werden allgemeine Empfehlungen zur PSNV von Kindern vorgestellt. Hierbei werden unterschiedliche Schwerpunkte der PSNV im Zeitverlauf dargestellt und Herausforderungen in der Versorgung aufgezeigt.

## Kinder als besondere Zielgruppe innerhalb der Psychosozialen Notfallversorgung (PSNV)

### Grundsätzliche Bedeutung von Bezugspersonen

Die Kindheit ist eine Zeit zentraler Entwicklungen. Konzepte über die eigene Person, die Welt und die eigene Person in dieser Welt werden zumeist über soziale Lernerfahrungen erworben [8]. Dabei beeinflussen Bindungspersonen die kindliche Entwicklung nicht nur über Erklärung und Wissensvermittlung. Gerade bei jungen Kindern haben sie die Aufgabe, deren Gefühle aktiv zu regulieren, bis diese zunächst noch mit äußerer Hilfe, später auch zunehmend selbst in der Lage sind, eigene Affekte zu steuern [7]. Der Inhalt kindlicher kognitiver Konzepte unterscheidet sich teils deutlich von den Vorstellungen erwachsener Personen und wird erst durch Erfahrung erworben. Das erwachsene Todesverständnis lässt sich beispielsweise neben dem Verständnis der eigenen Sterblichkeit über 4 Subkonzepte definieren. Während bereits recht früh verstanden wird, 1) dass alle Menschen sterben müssen (Universalität) und dass 2) der Tod irreversibel ist, werden Verstehenskonzepte wie 3) die Beendigung körperlicher Funktion mit dem Tod oder 4) die Kausalität des Versterbens erst später entwickelt [9]. Je nachdem, ob und wie diese Konzepte beim Kind bereits entwickelt wurden, verändert sich die Art und Weise, wie es die Konfrontation mit Tod oder Lebensgefahr verarbeitet.

Bindungstheoretische Modelle postulieren einen grundlegenden Einfluss der Interaktion mit vertrauten Menschen auf die Möglichkeit von Kindern und Heranwachsenden zu lernen, herausfordernde Erfahrungen zu integrieren und sich zu entwickeln. Eine beispielhafte klinische Anwendung dieser Befunde ist das Modell „Kreis der Sicherheit“ [10]. Demnach kommen dem Verhalten der Erwachsenen vereinfacht gesehen 2 unterschiedliche Funktionen zu. Einerseits soll es das Sammeln neuer Erfahrungen, Explorationsverhalten und folglich eine „in die Welt gerichtete“ Wegbewegung des Kindes von den vertrauten Strukturen ermöglichen. Im Falle heraus- oder überfordernder Erfahrungen soll dagegen eine Rückbewegung in die vertrauten Strukturen unterstützt werden. Die hier erfahrene Sicherheit, die unterstützende Orientierung und Beruhigung können wiederum als Voraussetzung für neuerliches Explorieren, Auseinandersetzen und Wachstum gesehen werden [7]. Cooper et al. [11] formulieren, dass beinahe alles, was man über die Unterstützung des (kindlichen) Sicherheitserlebens wissen muss, Folgendes sei: *Sei immer größer, stärker, weiser und freundlich. Wann auch immer möglich, folge meinen kindlichen Bedürfnissen. Wann immer notwendig, geh in Führung.*

**Figure 1:**
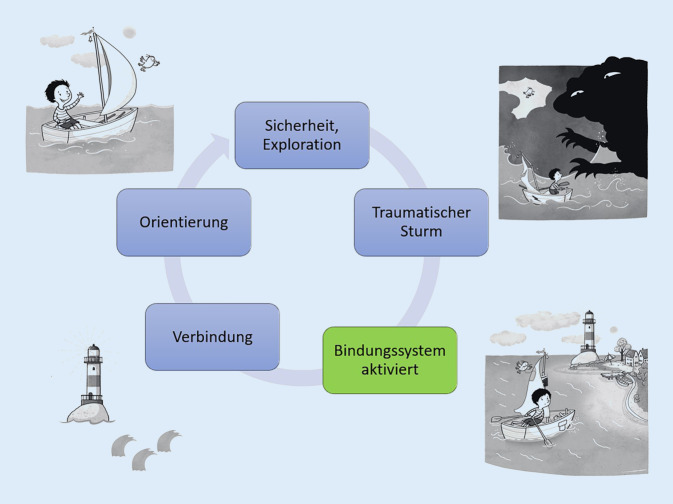

Eine Anwendung des bindungstheoretischen Modells „Kreis der Sicherheit“ zum Zusammenwirken von Bezugspersonenverhalten und kindlicher Verarbeitung auf den Bereich kritischer Lebenserfahrungen von Kindern [12] zeigt Abb. 1. In diesem vereinfachenden psychoedukativen Bild für Bezugspersonen orientieren sich Kinder als kleine Seefahrende nach der Konfrontation mit potenziell traumatisierenden Situationen (Sturm) in Richtung ihres bereits vertrauten und sicherheitsspendenden Hafens. Bindungspersonen (Leuchttürmen) kommt die Aufgabe zu, mittels Verbindung zu den Kindern und orientierender und erklärender Impulse eine verarbeitende Auseinandersetzung mit der hoch belastenden Situation zu unterstützen.

Die elterlichen Kompetenzen im Umgang mit ihren Kindern haben nicht nur für den Umgang mit Herausforderungen und die allgemeine Entwicklung eine besondere Bedeutung, sondern auch für die Verarbeitungsmöglichkeiten von hoch belastenden Lebenserfahrungen. In der Literatur ist das Erleben oder Fehlen sozialer Unterstützung als Faktor für die Verarbeitung potenziell traumatisierender Ereignisse wiederholt belegt worden [13–16]. Eltern spielen hierbei zumeist eine zentrale Rolle [17]. Im Folgenden soll ein differenzierter Überblick über ausgewählte Forschungsergebnisse gegeben werden. Verschiedene Möglichkeiten des Elternverhaltens und deren jeweilige Bedeutung für die Verarbeitung eines erlebten Notfalls beim Kind werden darstellt. Hierauf aufbauend werden Implikationen für die PSNV von Kindern zusammengefasst.

### Bedeutung des Bezugspersonenverhaltens für die Verarbeitung von Notfallereignissen

Zur Veranschaulichung der Bedeutung des Bezugspersonenverhaltens für die kindlichen Möglichkeiten, hoch belastende Erfahrungen zu verarbeiten, wird im Folgenden exemplarisch eine typische Einsatzindikation der PSNV dargestellt: die Betreuung Angehöriger nach Suizid im Nahfeld.

Laut Weltgesundheitsorganisation (WHO) liegt in Ländern mit einem hohen durchschnittlichen Haushaltseinkommen wie in Deutschland die Prävalenz von Suizidversuchen bei 3 pro 1000 erwachsenen Einwohner*innen [18]. Im Jahr 2020 verstarben in Deutschland insgesamt 9206 Personen durch Suizid [19]. Das waren über 25 offiziell als Suizid gewertete Todesfälle pro Tag. Die Anzahl der Suizidversuche übersteigt nach Schätzungen der Deutschen Depressionshilfe die der Suizide um den Faktor 15–20 [20], wobei jeweils mehrere Angehörige durch die Folgen betroffen sind. Diagnoseleitlinien definieren auch ohne direkte Zeugenschaft allein die Überbringung der Nachricht, dass ein nahes Familienmitglied von einem traumatischen Ereignis wie unfall- oder gewaltbedingter Verletzung oder Tod betroffen ist, als mögliches Auslöseereignis für die Entwicklung einer posttraumatischen Belastungsstörung [21]. Die unmittelbare Unterstützung des Umfeldes nach Suizid oder Suizidversuch stellt folglich eine indizierte und häufige Indikation in der PSNV dar.

Für die hier ausgewählte Indexsituation wurden das Geschlecht des Opfers, die Todesart und weitere Aspekte anhand statistischer Häufungen ausgewählt [19]:

Beim Betreten seines Kinderzimmers findet der zehnjährige Sohn den Familienvater (44 Jahre) auf, der sich mit einem Elektrokabel am Hochbett des Sohnes erhängt hat. Um seinem Vater zu helfen, versucht der Sohn ihn anzuheben, während er um Hilfe ruft. Die Mutter (38 Jahre) und das weitere Kind der Familie (weiblich, 6 Jahre), das im Wohnzimmer gespielt hat, kommen hinzu. Im Folgenden werden Rettungsdienst, Polizei und PSNV alarmiert. Die eingeleiteten Reanimationsbemühungen im Kinderzimmer verlaufen frustran.

Auch unabhängig von einer direkten Bezeugung sind Kinder, die wie in diesem Beispiel vom Suizid einer Bezugsperson betroffen sind, in besonderer Weise herausgefordert [22, 23]. Sie weisen ein erhöhtes Risiko für die Entwicklung psychischer Erkrankungen auf [24]. Zusätzlich ist das Risiko betroffener Kinder und Jugendlicher, selbst im weiteren Lebensverlauf suizidale Handlungen zu vollziehen, signifikant erhöht [25, 26]: Im Vergleich zu Kindern, die ein Elternteil bei einem Unfall verloren haben, ist dieses Risiko bei suizidbetroffenen Kindern um 82 % erhöht [25]. Besonders gefährdet sind Heranwachsende, die beim Suizid des Elternteils unter 6 Jahre alt waren [24]. Eine frühe Versorgung betroffener Menschen nach dieser Art von Ereignissen wird empfohlen [27].

In der Anwendung des o. g. allgemeinen bindungstheoretischen Modells scheint die Unterstützung der kindlichen Verarbeitung durch die Mutter von verschiedenen Aspekten abhängig zu sein, es stellen sich Fragen wie: Ist die Mutter als verbleibende zentrale Bindungsperson trotz eigener Belastung in der Lage, feinfühlig kindliche Bedürfnisse wahrzunehmen? Erkennt sie oder, falls vorhanden, eine andere vertraute Bezugsperson eine mögliche entstehende kindliche Symptombelastung in den Folgetagen? Können drängende kindliche Fragen und Reaktionen, wie bspw. Angst oder sich aufdrängendes Wiedererleben (Intrusionen) durch den engen Körperkontakt mit dem Verstorbenen, begleitet oder reguliert werden oder belasten sie die Bezugsperson selbst so stark, dass sie bei Sichtbarwerden zu einer Störung der Eltern-Kind-Interaktion führen? Welche Bewältigungsstrategie (Copingverhalten) der Kinder wird verstärkt oder durch die Mutter modelliert? Verfügt sie über ausreichende eigene Verarbeitungsmöglichkeiten?

Stover et al. [28] veröffentlichten eine Untersuchung, die der Frage nach der Übereinstimmung elterlicher und kindlicher Einschätzungen von Traumaerleben und Erleben von Belastungssymptomen nachging. 76 Kinder und Jugendliche (7–17 Jahre), die selbst potenziell traumatisierende Ereignisse erlebt hatten, wurden über die Traumasektion am Yale Child Study Center rekrutiert. Die Zuweisung erfolgte über die Polizei, ein Krankenhausprogramm oder eine kinderärztliche Notaufnahme. Eine durchgängige Übereinstimmung der Einschätzungen von Bezugspersonen und Kind konnte nicht belegt werden. Insbesondere die Beschreibungen der Auswirkungen des Erlebten auf die Kinder korrelierten weder zum Ereigniszeitpunkt noch zum späteren Erhebungszeitpunkt signifikant miteinander. Ebenfalls nur schwache Übereinstimmungen ergaben sich beim Erkennen früher klinischer Traumasymptome, insbesondere in den Bereichen Vermeidungsverhalten und Übererregung.

Die Forschergruppe diskutierte mögliche Erklärungen für diese Ergebnisse. So sei es eine Möglichkeit, dass Erwachsene über bestimmte Erlebnisse kaum oder gar nicht sprechen. Gründe hierfür könnten eigene Unsicherheit oder Betroffenheit oder der Wunsch sein, Kinder oder sich selbst zu schützen. Aus der Perspektive der PSNV ergibt sich hieraus für die Folgezeit nach hoch belastenden Erfahrungen eine bedeutsame Herausforderung. Sollten Eltern Belastungsfolgen bei Kindern nicht erkennen oder nicht um die Bedeutung der sichtbaren Symptome wissen, hat dies Einfluss auf die Nutzung innerfamiliärer und außerfamiliärer sowie professioneller Hilfe und den Zugang dazu. Folglich stellt die frühe Sensibilisierung des Bezugssystems ein wichtiges PSNV-Ziel dar.

Bezugspersonen haben nicht nur in der Einschätzung von Hilfebedarf und der Auswirkung des Erlebten auf ihre Kinder eine relevante Rolle in der kindlichen Traumaverarbeitung. In einer Metaanalyse untersuchten Williamson et al. [29], inwiefern Elternverhalten einen Prädiktor für die kindliche posttraumatische Symptomschwere darstellt. Es zeigten sich Zusammenhänge des elterlichen Verhaltens mit der kindlichen Symptomschwere insofern, dass negatives Verhalten (bspw. Überbehütung) mit einer größeren Symptomschwere und positives Verhalten (bspw. Unterstützung) mit einer geringeren Symptomschwere korrelierte.

Zur Wirkrichtung dieser Befunde äußerten Hiller et al. verschiedene Hypothesen [30]. So könne überbehütendes Elternverhalten, wie in der allgemeinen Literatur zu kindlichen Angststörungen beschrieben, eine Reaktion der Bezugspersonen auf die kindliche Traumabelastung und nicht deren Ursache sein [31]. Ergebnisse, die sich mit einer unidirektionalen Wirkrichtung der Eltern in Richtung der Kinder nicht ohne Weiteres vereinbaren lassen, stellen auch Scheeringa et al. [32] in einer prospektiven zweijährigen Studie mit Müttern und Kindern (1–6 Jahre) heraus. Eine zu Erfahrungen in der Einsatzpraxis passende mögliche Erklärung für diese Befunde ist, dass sich Bezugspersonen durch die Konfrontation mit den kindlichen Symptomen und/oder einen Mangel an erlebter Handlungskompetenz überfordert und hilflos fühlen und deshalb Vermeidungsverhalten zeigen. Dieser und auch die folgenden Befunde unterstreichen die Erweiterung der rettungsdienstlichen Versorgungslogik in der PSNV von Kindern: Erfolgt die medizinische Behandlung eines körperlich verletzten Kindes naturgemäß direkt am Kind, so stehen bei der notfallpsychologischen Versorgung eines unverletzten Kindes Eltern und Bezugspersonen ebenfalls im direkten Fokus der Versorgung.

In einer auf die o. g. Befunde folgenden Längsschnittstudie untersuchten Hiller et al., wie sich das Verhalten der Bezugspersonen auf die frühe Notfallverarbeitung von Kindern auswirkt [30]. Hierzu befragten sie 132 Kind-Elternteil-Dyaden, die sie über die Notaufnahmen von 4 Krankenhäusern rekrutierten. Mittels objektiver und Selbstbeschreibungsmaße fanden Erhebungen 1, 3 und 6 Monate nach dem von den Kindern (6–13 Jahre) erlebten Indexereignis statt. In ihrer Studie konnten sie zeigen, dass auch unter Kontrolle der initialen kindlichen Symptome negative elterliche Bewertungen zu einem frühen Zeitpunkt ein starker Prädiktor für die kindliche posttraumatische Symptomschwere nach 6 Monaten war. Sowohl über Selbstberichte als auch über beobachtete Eltern-Kind-Interaktion ließen sich negative Auswirkungen dieser Erwachsenenkognitionen nachweisen. Über 3 Aspekte der Bewertungen von Bezugspersonen war es möglich, die kindliche Symptomschwere nach 6 Monaten vorherzusagen: 1) die elterlichen Bewertungen in Richtung einer wahrgenommenen Verletzlichkeit oder einer andauernden Beeinträchtigung des Kindes, 2) die elterliche Fokussierung auf die negativen Aspekte des Ereignisses (bspw. wie gefährdet das Kind war, wie bedeutsam die Bedrohung war) und 3) das elterliche Erleben von (Selbst‑)Beschuldigung. Da in den untersuchten Fällen die initiale Belastung statistisch kontrolliert wurde, konnten Belege dafür gefunden werden, dass das Verhalten und die Kognitionen der Bindungspersonen die kindliche Ereignisverarbeitung und die Entwicklung der kindlichen Bewertungen beeinflussen.

Ähnlich wie im eingangs beschriebenen allgemeinen Bindungsmodell verweisen die Forschenden darauf, dass Kinder auf eine Orientierung an den und durch die Bezugspersonen angewiesen sind, um neuartige, bedrohliche oder komplexe Situationen einordnen zu können. In diesem Sinne können Eltern nicht nur ermutigend, sondern auch erschwerend auf die Entwicklung kindlicher Konzepte zu Ereignissen und deren Bedeutung für die eigene Person und Sicherheit einwirken [33]. Ein weiteres Ergebnis der Forschung von Hiller et al. [30] war, dass die Art und Weise, wie Erwachsene ihre Kinder zum Umgang mit der erlebten Situation anleiteten, ein Prädiktor für die spätere Entwicklung der kindlichen Traumaverarbeitung war. Wurden die Kinder durch die Eltern ermutigt bspw. Orte oder Personen zu meiden, die an das Indexereignis erinnern könnten, ließ sich hierdurch eine spätere stärkere Symptomschwere bei den betroffenen Kindern vorhersagen.

Die Möglichkeiten von Kindern, erlebte bedrohliche Situationen zu verarbeiten, stehen in der Literatur ebenfalls in einem Zusammenhang mit dem Belastungsgrad ihrer Bindungspersonen. Leiden Eltern postsituativ selbst an einer PTBS, ist dies als Risikofaktor für die kindliche Verarbeitung zu werten [34]. Ebenso zeigen sich Zusammenhänge zwischen erwachsenem Copingverhalten und kindlicher Verarbeitung. Dies gilt nicht nur für die Auswirkungen elterlicher Bewertung oder Vermeidung. Beispielsweise berichteten Eltern, die selbst eher maladaptive Copingstrategien zur Bewältigung von Notsituationen einsetzten, häufiger, körperliche Züchtigung anzuwenden, was mit einem erhöhten Risiko für PTBS bei Kindern sowohl kurz- als auch mittelfristig einhergeht [35, 36].

Auch wenn in diesem Beitrag der Schwerpunkt auf der Forschungslage zur Auswirkung elterlichen Verhaltens auf die kindliche Verarbeitung von Notfallereignissen liegt, wird dieser Prozess oft durch weitere Bezugspersonen beeinflusst, wie beispielsweise Karutz et al. (2021) darstellen [37]. Erwähnenswert ist hier die mögliche Unterstützung durch die Gruppe von Gleichaltrigen auf privater oder Lehrkräfte auf professioneller Seite. Neben Belegen für die schützende Wirkung dieser Gruppen schilderte ein Teil der befragten Jugendlichen, dass Schule nach dem Erleben eines Notfallereignisses nicht nur als Entlastungs-, sondern teilweise auch als zusätzlicher Belastungsfaktor erlebt wurde [38].

## Allgemeine Empfehlungen und Herausforderungen in der Psychosozialen Notfallversorgung von Kindern

### Grundgedanke und Zielsetzung der PSNV

Das Feld der PSNV ist durch eine hohe Begriffsvielfalt geprägt, die mit der inhaltlichen Spezifizierung des Begriffs „Psychosoziale Notfallversorgung“ im Rahmen einer Konsensus-Konferenz systematisiert wurde [38]. Die konsentierte Definition des Oberbegriffes lautet:

Der Begriff Psychosoziale Notfallversorgung (PSNV) beinhaltet die Gesamtstruktur und die Maßnahmen der Prävention sowie der kurz-, mittel- und langfristigen Versorgung im Kontext von belastenden Notfällen bzw. Einsatzsituationen.

Die PSNV beinhaltet folglich heterogene Angebote von der einmaligen Psychosozialen Akuthilfe über allgemeine und spezialisierte Beratungsangebote bis hin zu ambulanten und stationären heilkundlichen Maßnahmen der Regelversorgung.

Auch das Feld der Anbietenden und die Trägerlandschaft gestalten sich vielfältig und umfassen Laienhilfsangebote in Trägerschaft von Hilfsorganisationen oder Kirchen, gemeinnützige und wirtschaftliche Anbietende. Dazu kommen notfallversorgende Angebote innerhalb bestehender Systeme, wie bspw. in Schulen oder Betrieben, die teilweise im Bereich sozialrechtlicher Versorgungsansprüche oder bei privaten Versicherungen des betroffenen Unternehmens (bspw. Banken) angesiedelt sind.

Als übergreifende Ziele der PSNV werden die „Prävention von psychosozialen Belastungsfolgen“, die „Früherkennung von psychosozialen Belastungsfolgen nach belastenden Notfällen bzw. Einsatzsituationen“ und die „Bereitstellung von adäquater Unterstützung und Hilfe für betroffene Personen und Gruppen zur Erfahrungsverarbeitung sowie die angemessene Behandlung von Traumafolgestörungen ...“ genannt [38].

Angesichts dieser Vielfalt und Heterogenität kommt der differenzierten Betrachtung von PSNV-Aufgaben im Zeitverlauf eine besondere Bedeutung zu.

### Ansätze der PSNV im Zeitverlauf

**Figure 2:**
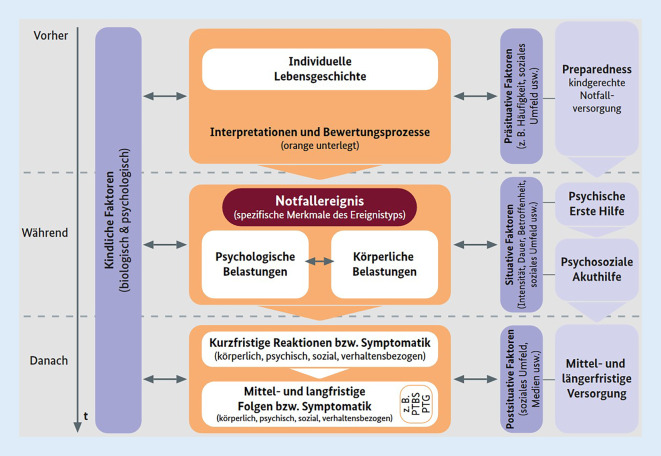

Die Verarbeitung hoch belastender Lebensereignisse gestaltet sich über die Zeit hinweg unterschiedlich. Stehen unmittelbar Themen wie initiales Realisieren des Geschehenen oder Erleben von Sicherheit im Vordergrund, folgen Aspekte wie Klärung von Verantwortlichkeiten oder Beachtung spezifischer Traumafolgen meist erst später [27]. Auch notfallversorgende Angebote gestalten sich verlaufsabhängig. Je nach Zeitpunkt wird zwischen akuten Notfallinterventionen und spezifischen Frühinterventionen unterschieden und es werden verschiedene Handlungsziele für die jeweiligen Phasen definiert ([27]; Abb. 2).

Hobfoll et al. formulieren in einer viel zitierten Arbeit (ohne besonderen Fokus auf die Situation von Kindern) 5 allgemeine, empirisch untermauerte Interventionsprinzipien für die unmittelbare bis mittlere Hilfe: 1) Förderung eines Gefühls der Sicherheit, 2) Beruhigung, 3) Förderung eines Gefühls der Selbst- und Gemeinschaftswirksamkeit, 4) Verbundenheit und 5) Hoffnung [39]. Für die PSNV von Kindern gelten ähnliche Grundsätze, die auf ihre Situation und Bedürfnisse angepasst sind. Karutz [40] nennt das 1) Wahrnehmen alters- und situationsspezifischer Bedürfnisse, 2) Vermitteln verständnisfördernder Informationen, 3) die Stärkung der Selbstwirksamkeit, 4) den Kontakt zu vertrauten Bezugspersonen und 5) die Vermittlung von Schutz und Sicherheit.

Bei der Psychischen Ersten Hilfe und der Psychosozialen Akuthilfe liegt der Fokus zunächst auf der Erfüllung grundlegender Bedürfnisse, der Herstellung von Sicherheit und der Stabilisierung der betroffenen Kinder [37]. Die National Commission on Children and Disasters (NCTSN; [41]) gibt dafür generelle Empfehlungen, wie z. B. das Einnehmen der Augenhöhe von Kindern, Zuhören, Unterstützung bei der Verbalisierung von Gefühlen, die Vermittlung verständlicher Informationen und der Einbezug von Bezugspersonen. Weitere Empfehlungen beinhalten, Kinder nicht alleine zu lassen, (vorsichtig) Körperkontakt herzustellen, Kuscheltiere zu nutzen, auf Fragen möglichst offen und ehrlich zu antworten, Reaktionen zu legitimieren und für Ablenkung zu sorgen [37].

Das Ziel von Frühinterventionen, die im Zeitverlauf später als Notfallinterventionen verortet werden, ist, das Geschehene begreiflich zu machen und die Entwicklung von Traumafolgestörungen zu verhindern bzw. gefährdete Kinder zu erkennen und einer geeigneten Behandlung zuzuführen [27]. Dabei ist die Ausbildung einer Traumafolgestörung nicht als monokausale Folge eines hoch belastenden Lebensereignisses zu verstehen. Die individuelle Verarbeitung hängt vielmehr von prä-, peri- und posttraumatischen Faktoren, der Art des Ereignisses und den (Vor‑)Erfahrungen ab [5]. Es gibt Hinweise, dass der frühen Folgezeit nach einem Notfall bei Kindern und Jugendlichen dabei eine besondere Bedeutung zukommt, da Risikofaktoren in dieser Zeit einen stärkeren Einfluss auf die Verarbeitung und damit auf die Entstehung einer Traumafolgestörung zu haben scheinen als prätraumatische Faktoren [16]. Insbesondere sozialer Rückzug, schlechtes Funktionsniveau der Familie, Ablenkung und Gedankenunterdrückung sowie mangelnde soziale Unterstützung [16, 34] stehen im Zusammenhang mit ungünstigen Verarbeitungsprozessen und der Entstehung einer PTBS bei Kindern [16]. Darüber hinaus haben die dysfunktionale Ereignisbewertung durch das Kind selbst sowie die postsituative Verfügbarkeit von Bezugspersonen und deren Verhalten Einfluss auf die Traumaverarbeitung [16, 29]. Somit ist die PSNV auch im Nachgang zur unmittelbaren Psychosozialen Akuthilfe für Kinder dringend indiziert.

### Herausforderungen in der PSNV von Kindern

Wie bereits aufgezeigt, ist es von hoher Bedeutung nach der Psychosozialen Akuthilfe weitere Angebote vorzuhalten, um bei Bedarf auch über eine einmalige Akutberatung hinaus Einfluss auf die kindliche Verarbeitung nach einem Notfallgeschehen nehmen zu können. Ob und wie spätere Hilfsangebote in Anspruch genommen werden, entscheidet sich vielfach an den Anforderungen im Zugang [42]. Ob ein Hilfsangebot niedrig- oder hochschwellig ist, hängt von den Zugangsbedingungen auf unterschiedlichen Ebenen ab, die räumlich, zeitlich, administrativ, monetär, kompetenzorientiert, lebenslagenorientiert, psychologisch sowie strukturell und rechtlich sein können [43]. Strukturelle und persönliche Barrieren, die nicht zuletzt in den heterogenen Versorgungsstrukturen Deutschlands begründet liegen, stellen Hinderungsgründe für die Weiterversorgung belasteter Betroffener dar [44].

Insbesondere in ländlichen Regionen Deutschlands gibt es wenige Kinder- und Jugendpsychotherapeut*innen in der ambulanten Versorgung und es kann zu langen Wartezeiten für Betroffene kommen [45]. Darüber hinaus fehlen sowohl in der ärztlichen als auch in der psychologischen kinder- und jugendtherapeutischen Grundausbildung relevante Inhalte und der Anteil von Fachkräften mit traumaspezifischen Zusatzqualifikationen ist gering [44, 45]. Praxiserfahrung und aktuell laufende Forschung der Autor*innen weisen darauf hin, dass diese Befunde neben heilkundlichen auch für beratende Angebote gelten. Als wegweisende Faktoren für die Inanspruchnahme von Angeboten werden sozioökonomischer Status, Gesundheitsüberzeugungen, familiäre Einflüsse und nicht zuletzt der wahrgenommene Bedarf, sowohl durch das Kind als auch durch die Bezugspersonen, genannt [44]. Die Abhängigkeit minderjähriger Kinder stellt eine weitere Hürde in der Versorgung dar, da sie nur begrenzt eigenständig entscheiden und einen Wunsch nach Inanspruchnahme von Hilfe umsetzen können. Bezugspersonen stellen hier ein Nadelöhr dar, da sie, bevor Kinder nötige Hilfe erhalten, körperliche, psychische und/oder soziale Auffälligkeiten beim Kind wahrnehmen und adäquat darauf reagieren müssen.

### Entwicklungen in der PSNV von Kindern

Kindern und ihren Bezugspersonen auch über die Psychosoziale Akuthilfe hinaus verarbeitungsfördernde Angebote zu machen, die nicht erst im hochschwelligen Bereich der heilkundlichen Versorgung und nach erfolgter Erkrankung ansetzen, ist gleichsam sinnvoll wie herausfordernd. Erst im vergangenen Jahr wies Karutz [46] hierzu auf *Das fehlende Bindeglied* der mittelfristigen Notfallversorgung für Kinder und Familien hin. Ähnliche Erfahrungen führten 2007 zur Entwicklung eines Konzepts der „Aufsuchenden Psychosozial-Systemischen Notfallversorgung“ (APSN; [47]). Stand betroffenen Kindern zum damaligen Zeitpunkt im Großraum München nach Notfällen Krisenintervention im Rettungsdienst oder Notfallseelsorge zur Verfügung, fehlten nach der einmaligen Akuthilfe weitere, auf Notfallverarbeitung spezialisierte PSNV-Unterstützungsmöglichkeiten. Erst im Falle einer verfestigten Pathologie gab es, häufig mit langen Wartezeiten verbundene, bspw. psychotherapeutische Angebote. Die APSN ist ein traumaspezifisches, nicht therapeutisches Beratungsangebot, das als Frühintervention nach potenziell traumatisierenden Lebensereignissen über die Dauer von bis zu einem Jahr präventiv, stabilisierend und den Verarbeitungsverlauf beobachtend tätig wird. Die dem Rettungsdienst ähnliche vorwiegend aufsuchende Arbeitsweise erfüllt dabei mehrere Zwecke. Zum einen schafft die gewohnte und sicherheitsstiftende Umgebung Orientierung. Zum anderen ermöglicht sie, in einer zumeist aufgewühlten und nicht selten auch organisatorisch belastenden Zeit (Polizei, Rechtsmedizin, Bestattung, Versicherungen, Behörden usw.), die Beratung von Kindern, Bezugspersonen und beteiligten Fachkräften ohne zusätzlichen Zeitaufwand.

Neben regionalen Modellprojekten gewinnt die PSNV von Kindern auch in Ausbildung und Lehre mehr Bedeutung. Sahen die vorangegangenen gemeinsamen Qualitätsstandards und Leitlinien zu Maßnahmen der PSNV im Bereich der Psychosozialen Akuthilfe [48] insgesamt 4 Unterrichtseinheiten für „besondere Zielgruppen“ vor, zu denen Kinder und Jugendliche, Senior*innen, von Krisensituationen Betroffene in Bildungs- und Betreuungseinrichtungen sowie Menschen mit Behinderungen gehörten, so erfolgte bei der ab Oktober 2022 verpflichtenden Neuerung des „Mindeststandards in der Psychosozialen Akuthilfe (PSAH)“ [49] eine Erweiterung bzgl. Inhalt und Umfang. Ähnlich verhält es sich in den Leitlinienentwicklungen zur „Diagnostik und Behandlung von akuten Folgen psychischer Traumatisierung“. Beinhaltete die S2-Leitlinie Diagnostik und Behandlung von akuten Folgen psychischer Traumatisierung von Flatten et al. [50] noch keine besondere Kinderperspektive, änderte sich hier Ende 2019 die Ausrichtung [27]. So heißt es in der handlungsleitenden Empfehlung Nr. 21: „Frühinterventionen für Kinder und Jugendliche mit einem erhöhten Risiko sollen dem Alter und dem Entwicklungsstatus angepasst sein und die Eltern bzw. die Bezugspersonen einbeziehen“ [27]. Und Kapitel 7.4 beschäftigt sich ausschließlich mit den Besonderheiten des Kindes- und Jugendalters.

## Fazit

Kinder befinden sich nach Notfällen in einer anderen Situation als Erwachsene. Zugleich hat das Verhalten erwachsener Bezugspersonen auf verschiedene Weise Einfluss auf die kindlichen Verarbeitungsmöglichkeiten und Erkrankungsrisiken. Psychosoziale Notfallversorgung (PSNV) sollte demzufolge einem systemischen Blick folgen und gezielt Kompetenzen, Verhaltensweisen und Belastungen Erwachsener ebenso wie deren Umgang mit kindlichen Belastungsreaktionen in die Bedarfsermittlung einbeziehen.

PSNV sollte mit verschiedenen Schwerpunkten über die Akuthilfe hinaus angeboten werden. Der oftmals hochschwellige Zugang und das Fehlen spezifischer Hilfen stellen hierbei eine Herausforderung dar.

## Footnotes

1 Der vorliegende Beitrag fokussiert die PSNV nach hoch belastenden Erfahrungen. Aus diesem Grund finden entwicklungspsychologisch und psychotraumatologisch relevante Konzepte aus verwandten Forschungsfeldern, wie beispielsweise die Auswirkung der Traumatisierungsart (z. B. Monotrauma vs. sequenzielles Trauma oder Traumatisierung durch Unfallfolgen vs. Übergriffe durch nahe Bezugspersonen) oder auch die Auswirkung der sogenannten ungünstigen Kindheitserfahrungen (Adverse Childhood Experiences), hier keine weitergehende Erwähnung.
